# AP1S1 missense mutations cause a congenital enteropathy via an epithelial barrier defect

**DOI:** 10.1007/s00439-020-02168-w

**Published:** 2020-04-18

**Authors:** Katharina M. C. Klee, Andreas R. Janecke, Hasret A. Civan, Štefan Rosipal, Peter Heinz-Erian, Lukas A. Huber, Thomas Müller, Georg F. Vogel

**Affiliations:** 1 Institute of Cell Biology, Biocenter, Medical University of Innsbruck, 6020 Innsbruck, Austria; 2 Department of Pediatrics I, Medical University of Innsbruck, Anichstrasse 35, 6020 Innsbruck, Austria; 3 Division of Human Genetics, Medical University of Innsbruck, 6020 Innsbruck, Austria; 4 Pediatric Gastroenterology, Bakirkor Training and Research Hospital, 34140 Istanbul, Turkey; 5 Pediatric Clinic of Preventive Medicine in Poprad, Jarmocna 27, 058 01 Poprad-Veľká, Slovakia

## Abstract

Congenital diarrheal disorders (CDD) comprise > 50 monogenic entities featuring chronic diarrhea of early-onset, including defects in nutrient and electrolyte absorption, enterocyte polarization, enteroendocrine cell differentiation, and epithelial integrity. Diarrhea is also a predominant symptom in many immunodeficiencies, congenital disorders of glycosylation, and in some defects of the vesicular sorting and transporting machinery. We set out to identify the etiology of an intractable diarrhea in 2 consanguineous families by whole-exome sequencing, and identified two novel *AP1S1* mutations, c.269T>C (p.Leu90Pro) and c.346G>A (p.Glu116Lys). AP1S1 encodes the small subunit of the adaptor protein 1 complex (AP-1), which plays roles in clathrin coat-assembly and trafficking between trans-Golgi network, endosomes and the plasma membrane. An *AP1S1* knock-out (KO) of a CaCo2 intestinal cell line was generated to characterize intestinal *AP1S1* deficiency as well as identified mutations by stable expression in KO background. Morphology and prototype transporter protein distribution were comparable between parental and KO cells. We observed altered localization of tight-junction proteins ZO-1 and claudin 3, decreased transepithelial electrical resistance and an increased dextran permeability of the CaCo2-AP1S1-KO monolayer. In addition, lumen formation in 3D cultures of these cells was abnormal. Re-expression of wild-type *AP1S1* in CaCo2-AP1S1-KO cells reverted these abnormalities, while expression of *AP1S1* containing either missense mutation did not. Our data indicate that loss of *AP1S1* function causes an intestinal epithelial barrier defect, and that *AP1S1* mutations can cause a non-syndromic form of congenital diarrhea, whereas 2 reported truncating *AP1S1* mutations caused MEDNIK syndrome, characterized by mental retardation, enteropathy, deafness, neuropathy, ichthyosis, and keratodermia.

**Electronic supplementary material:**

The online version of this article (10.1007/s00439-020-02168-w) contains supplementary material, which is available to authorized users.

## Introduction

Congenital diarrheal disorders (CDD) generally result from specific genetic defects inherited as autosomal recessive traits, with a severe clinical presentation. CDD are generally associated with feeding intolerance and malabsorption, and require major dietary interventions and most often parenteral nutrition, to sustain electrolyte and nutrient balance, and appropriate growth. Many different disorders can present with diarrhea as the first or predominant symptom in neonates (Canani et al. 2015; Thiagarajah et al. 2018). For instance, CDD affect intestinal epithelial and immune function, and these include deficiencies of proteins necessary for nutrient absorption such as lactase, where undigested lactose in the intestine causes osmotic diarrhea (Swallow 2003) and Na^+^/glucose cotransporter SLC5A1 (Turk et al. 1991), where resorption of galactose in the intestine is deficient and causes osmotic diarrhea, deficiencies for ion absorption and pH regulation such as chlorid/bicarbonate exchanger SLC26A3 (Aichbichler et al. 1997), for enterocyte polarization such as the motor protein myosin Vb (Muller et al. 2008), for enteroendocrine cell differentiation such as the transcription factor NEUROG3 (Wang et al. 2006), and epithelial integrity such as cell adhesion molecule EpCAM (Sivagnanam et al. 2008). Interestingly, early-onset diarrhea is also a prominent symptom of many types of immunodeficiency (Bennett et al. 2001) and of systemic metabolic diseases, like congenital glycosylation disorders and cystic fibrosis (Barrett 2000; Grünewald et al. 2002). Early-onset and persistent diarrhea also occur in systemic disorders resulting from defects in widely expressed components of the intracellular vesicular transporting and sorting machinery, such as Arthrogryposis, Renal dysfunction, Cholestasis (ARC) syndrome (Taha et al. 2007) and mental retardation, enteropathy, deafness, neuropathy, ichthyosis, keratodermia (MEDNIK) syndrome (Montpetit et al. 2008; Saba et al. 2005). MEDNIK syndrome, also known as “syndrome de Kamouraska” (syndrome from Kamouraska), is a genetic disorder that is caused by mutations in adaptor related protein complex 1 subunit sigma 1 (*AP1S1*) and beta 1 (*AP1B1*) genes (Alsaif et al. 2019; Martinelli and Dionisi-Vici 2014). Diagnostic procedures for these disorders and therapeutic pathways for the care of affected patients have only recently been suggested (Thiagarajah et al. 2018). The underlying genetic basis and pathophysiology of these disorders has been revolutionized by the availability of next-generation sequencing. This has led to the continued identification of new congenital diarrheas and enteropathies (O'Connell et al. 2018; Ozen et al. 2017; van Rijn et al. 2018).

Here we report the identification of two novel, homozygous *AP1S1* mutations, c.269T>C (p.Leu90Pro) and c.346G>A (p.Glu116Lys), in 3 patients with isolated, i.e., non-syndromic congenital intestinal failure. Deletion of *AP1S1* in a genome edited human intestinal epithelial cell line disrupted epithelial barrier function. Importantly, in contrast to wild-type *AP1S1*, re-expression of the here identified homozygous *AP1S1* mutations could not rescue the phenotype.

## Materials and methods

### Patients

There is a diagnostic and research focus on inherited diarrheas at the Department of Pediatrics I, Medical University of Innsbruck. Patients and patient samples are referred continuously for genetic testing to determine or confirm a suspected etiology of the disorder. Written informed consent for molecular research investigations was obtained from the patient’s parents, and the studies are approved by the local ethics committee (votum no. AN2016-0029 359/4.5). At our center, three patients with congenital diarrhea from two unrelated families were identified with *AP1S1* variants by whole-exome sequencing (WES).

### Molecular genetic studies

**Fig. 1:**
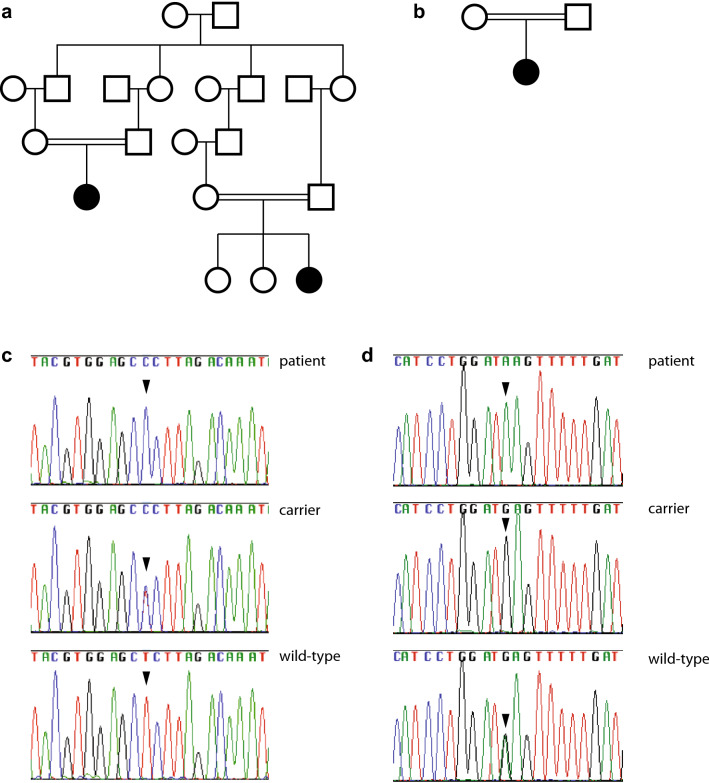
Identification of novel *AP1S1* missense mutations. Pedigrees of patients 1 and 2 (**a**) and patient 3 (**b**). Detection of *AP1S1* variants (**c**) c.269T>C; representative sequence traces are shown from patient 1 (patient), her mother (carrier) and from a control and **d** c.346G>A; representative sequence traces are shown from patient 3 (patient), her mother (carrier) and from a control

Genomic DNA was isolated from peripheral blood leukocytes by standard procedures. DNA samples from the patients, their healthy parents, and two healthy sibs (Fig. 1a–d) were genotyped with high-resolution single nucleotide polymorphism arrays (HumanCytoSNP-12v2 BeadChip SNP array, Illumina) interrogating 299,140 markers, according to the manufacturer’s instructions. Raw SNP call data were processed with the Genotyping Analysis Module of GenomeStudio 1.6.3 (Illumina). Copy-number variants and segments of loss-of-heterozygosity (LOH) were called and visualized using Nexus software and the SNPFASST segmentation algorithm (BioDiscovery Inc.). Multipoint likelihood-of-the-odds (LOD) scores were obtained with the Merlin program (Abecasis et al. 2002) under the hypothesis of an autosomal-recessive, fully penetrant mutation, inherited identical-by-descent. WES was performed in the mother of patient 1, as sufficient DNA was not available from any of the patients. Agilent's SureSelectXT2 V5 and V6 enrichment kits, respectively, and an Illumina HiSeq4000 instrument were used to generate 125-bp and 150-bp paired-end reads that were aligned to the human reference genome with Burrows–Wheeler transformation (Li and Durbin 2009). Polymerase chain reaction (PCR) duplicates were removed with PICARD (https://picard.sourceforge.net) and single nucleotide substitutions, and small indels were called with SAMtools software. All variants were submitted to SeattleSeq (https://snp.gs.washington.edu/SeattleSeqAnnotation/) for annotation, categorization, and filtering against public variant databases.

The AP1S1 variant designation is based on the NCBI reference sequence for transcript NM_001283.3 (corresponding to Ensembl transcript reference sequence ENST00000337619.9) and the genomic reference sequence NG_033082.1 (corresponding to Ensembl gene ENSG00000106367). The exon numbering is based on NG_033082.1. Nucleotide numbering uses + 1 as the A of the ATG translation initiation codon in the reference sequence, with the initiation codon as codon 1.

### Antibodies and reagents

Primary antibodies directed against beta-actin (WB, 1:2000, A2228, Sigma-Aldrich), AP1S1 (Western blotting (WB), 1:1000; ab217055; Abcam), claudin 3 (WB, 1:1000, (Immunofluorescence microscopy (IF), 1:200, SAB4500435, Sigma-Aldrich), E-cadherin (WB, 1:1000, IF, 1:200, 610181, BD Biosciences), HA (WB, 1:1000; IF, 1:500; MMS-101R; Covance) and ZO-1 (WB, 1:1000, IF, 1:200, 61-7300, Thermo Fisher) are commercially available. Primary a-tubulin (WB, 1:1000; 12G10) and DPPIV, IF, 1:100; HBB3/775/42) were acquired from the Developmental Studies Hybridoma Bank. Secondary horseradish-peroxidase-conjugated goat anti–mouse and goat anti-rabbit (1:5000; Sigma-Aldrich) were used for WB. Actin filaments were labeled with phalloidin–Alexa Fluor 568 (1:500; Life Technologies). Secondary Alexa Fluor-conjugated (Alexa Fluor 488 and 568) goat anti-mouse (1:1000; Life Technologies), goat anti-rabbit (1:1000; Life Technologies), and Hoechst 3342 (1:10,000; Thermo Fisher Scientific) were used for IF labeling.

### Cell lines, cell culture and genome editing

Hek293T and CaCo2 cells were cultured in DMEM (Sigma-Aldrich) containing high glucose, sodium pyruvate, 100 U/mL penicillin (Sigma-Aldrich), 100 µg/mL streptomycin (Sigma-Aldrich) and 10% FBS (Gibco) in a humidified atmosphere with 5% CO_2_ at 37 °C. For CRISPR/Cas9-mediated depletion, guide RNA (gRNA) targeting sequence for AP1S1 (5ʹ-GGTTCATGCTATTATTCAGC-3′) was selected using an online prediction tool [CHOPCHOP (Labun et al. 2016)]. The gRNA was cloned into a lentiCRISPRv2 vector via BsmBI restriction enzyme sites. lentiCRISPRv2 was a gift from F. Zhang [Massachusetts Institute of Technology, Cambridge, MA; Addgene plasmid 52961; (Sanjana et al. 2014)]. Lentiviral transduction was performed as described in the following paragraph. Depletion efficiency was verified via WB, and a gRNA-resistant cDNA was used for rescue experiments. One clone of parental CaCo2 cells was used for subsequent experiments. Colonies of transduced cells were selected by limiting dilution. Cells were cultured, subcultured and used for Western Blot experiments on 10 cm culture dishes (Sarsted, #83.3902). For experiments requiring fully polarized growth conditions [e.g., immunofluorescence experiments, transepithelial electrical resistance (TEER) and dextran permeability measurements], CaCo2 cells were seeded on 24-mm (Costar Transwell; pore size of 0.4 µm; Corning) and cultured for 14 days. CaCo2 3D cyst cultures were obtained essentially as described before (Vogel et al. 2015), briefly 5 × 10^4^/mL single cells were embeeded in Matrigel (BD Biosiences, #356231), plated on chamber slides and grown for 7 days.

### Measurement of TEER and Dextran permeability assay

Measurements of TEER were performed in CaCo2 cells of three independent experiments using the “STX2” Electrode in combination with the EVOM epithelial volt‐ohmmeter (World Precision Instruments). TEER measurements were performed on three different areas on the filter inserts and calculated as described previously (Thoeni et al. 2014). For monolayer permeability assays, low molecular weight dextran coupled to Texas-Red (10 kDa, Molecular Probes, Life Technologies) was added to the apical chamber of confluent polarized CaCo2 monolayers on Transwell filters (Costar Transwell; pore size of 0.4 µm; Corning) in a final concentration of 100 µg/mL and incubated for 10 h. Basolateral culture medium was taken after 10 h and measured in a luminometer (Tristar LB 941, Berthold Technologies, Germany) for Texas Red fluorescence.

### Plasmids and lentivirus production

Human *AP1S1* was amplified via PCR from cDNA and ligated into a pENTR-MCS-HA vector (a gift from S. Geley, Medical University of Innsbruck, Austria). Point mutations were introduced via PCR-based site-directed mutagenesis (AP1S1-T269C, AP1S1-G346A). For lentiviral transduction, cDNA was further subcloned via LR clonase into a pCCL-EFs-BlastiR-DEST lentiviral vector using Gateway Cloning Technology (Invitrogen). HS-NHE3 was previously described (Vogel et al. 2015).

Lentiviral plasmids were cotransfected with Lipofectamine LTX (Invitrogen) together with pVSV-G and psPAX2 in the Hek293LTV producer cell line. Viral supernatant was harvested 48 and 72 h after transfection and directly used for CaCo2 cell infection. 6 days after infection, cells were selected with 20 µg/mL puromycin (Sigma-Aldrich) or 20 µg/mL blasticidin S (Invitrogen).

### Western blotting

WB was performed essentially as described previously (Fialka et al. 1997). Polyvinylidene fluoride membranes were incubated with primary antibody at room temperature for 1 h or overnight at 4 °C. Secondary antibody incubation was performed for 1 h at room temperature. Chemiluminescence was exposed on films.

### Immunofluorescence microscopy (IF)

IF labeling of CaCo2 cells grown on Transwell filters and 3D cyst cultures were performed essentially as described previously (Vogel et al. 2015) and mounted in mowiol. Samples were analyzed at room temperature with an epifluorescence microscope (Axio Imager M1; Carl Zeiss) equipped with a charge-coupled device camera (SPOT Xplorer; Visitron Systems) and recorded with VisiView 2.0.3 (Visitron Systems). Objective lenses used were a 25 × oil immersion objective (numerical aperture of 0.8) and a 10 × air objective (numerical aperture of 0.3; Carl Zeiss). Single confocal planes or stacks were recorded with a confocal fluorescence microscope (SP5; Leica) using a glycerol 63 × lens with a numerical aperture of 1.3 (Leica) at room temperature and mounted in Mowiol. The recording software used was LASAF 2.7.3 (Leica). Images were deconvolved with Huygens Professional Deconvolution and Analysis Software (Scientific Volume Imaging), exported using Imaris 3D rendering (Bitplane), and adjusted for brightness, contrast, and pixel size.

### Software and statistics

The software used, if not already specified, were GIMP version 2.8.12 (GNU Image Manipulation Program; open source), ImageJ version 1.49a (National Institutes of Health), Photoshop CS6 (Adobe) and Illustrator CS6 (Adobe).

Dot box plot graphs were generated and the unpaired Mann–Whitney *U* test was calculated using R (version 3.6.2, https://www.r-project.org/) and ggplot2 package.

## Results

### Patient descriptions

Patient 1, a girl, was the first child born to healthy, consanguineous parents of Romani origin at 37 weeks of gestation. Birth weight and length were at the 5th and 3rd percentile. Prominent abdominal distension was due to dilated fluid-filled loops of intestine, and intractable diarrhea commenced after birth, independent of breastfeeding or formula initiation. There were increased bowel sounds and no meconium. The infant developed severe dehydration with metabolic acidosis and hyponatremia, and elevated stool sodium and chloride concentrations.

Several stool samples were analyzed during the first days of life: Na 107.5–120.0 mmol/L, K 8.1–9.5 mmol/L, Cl 81.1–85 mmol/L, pH 7.4–7.8. The child required total parenteral nutrition (TPN), but failed to thrive and died after 4 weeks of life. Neither ichthyosis, keratoderma, deafness nor dysmorphism were evident. Light microscopy of intestinal mucosa was unremarkable.

Patient 2, a girl, was born to another couple of healthy, consanguineous parents from the same Romani kindred as patient 1 and her parents (Fig. 1a). The clinical course was most similar to that of patient 1, with maternal polyhydramnios documented at the time of premature birth at 35 weeks of gestation. Weight and length at birth were at the 50th percentile. Intractable diarrhea was present from birth and rapidly led to severe dehydration. Laboratory findings were remarkable for hyponatremia and elevated stool sodium and chloride: Na 109.2–144.5 mmol/L (3–79 mmol/L), K 12.1–16.8 mmol/L (44–132 mmol/L), Cl 69.5–102.1 mmol/L (0–32 mmol/L), pH 7.2–8.0. The patient failed to thrive, and died at age 3 weeks of life. Patient 2 had two healthy older siblings, and another sib died due to congenital diarrhea at 9 days of life.

The non-syndromic form of congenital sodium diarrhea was considered as the likely diagnosis in this family.

Patient 3, is a female infant born after 37 weeks of gestation with a birth weight of 2660 g. Her healthy Turkish parents are first-degree cousins (Fig. 1b). Two previous pregnancies had ended in an abortus and with the postnatal death of a newborn, of unknown etiology. On postnatal day 8, patient 3 was referred for inadequate breastfeeding and diarrhea accompanied by weight loss (17% of birth weight), metabolic acidosis, an episode of myoclonic seizures, responsive to phenobarbiturate, and septic appearance. The diarrhea worsened with enteral feeding, and vomiting occurred. She was referred to a tertiary center for endoscopic evaluation and suspected malabsorption. Ad admission, she had elevated liver enzymes (ALT 109 U/L, AST 118 U/L) with normal coagulation parameters. She developed anemia without bleeding and responded to transfusions. Diarrhea persisted under TPN, and she failed to thrive with a weight below 2800 g at 2 months of age, with normal serum electrolyte levels. Fever and leukocytosis appeared on the 8th day of ICU hospitalization, and antibiotic treatment was started. *Serratia marcescens* was found in a blood culture suggesting a catheter infection and antimicrobial treatment was tailored accordingly. She died of septic shock on postnatal day 95. Light microscopy of intestinal mucosa was unremarkable; a form of autosomal-recessive secretory diarrhea was suspected.

### Disease locus mapping and detection of a novel *AP1S1* variant in patients 1 and 2

At the time of referral of patients 1 and 2, the disease genes for the non-syndromic form of congenital sodium diarrhea were not known, and a genome-wide autozygosity mapping was initiated in the Romani kindred, that revealed a single chromosomal region, which was significantly linked with the disease locus. This region spanned 4-Mb on chromosome 7q22.1. There were scarce amounts of DNA from patients 1 and 2 left, however, and WES was subsequently performed in the DNA sample from patient 1’s mother. Unexpectedly, WES identified a heterozygous *AP1S1* variant c.269T>C causing a leucine to proline change at codon 90 (g.100800744T>C; p.(Leu90Pro)) within the linked chromosomal region (Fig. 1c). There were no other rare or private variants seen within coding regions or at splice sites within this linkage interval. Follow-up Sanger sequencing demonstrated segregation of this variant with the disease, i.e., this variant was present in homozygous state in both patients, and heterozygous in their four parents, and two healthy sibs of patient 2. The phenotype and variant information were submitted to the LOVD database (https://databases.lovd.nl/shared/genes/AP1S1). This variant was not listed in dbSNP, ESP, and EXAC databases, and was predicted to damage protein function by 5 algorithms: Polyphen2: probably damaging (score 1.00, range 0–1), SIFT: not tolerated (score 0.00, range 1–0), CADD: pathogenic (score 22.5, range 0–100), PROVEAN: deleterious (score − 6.588), MutationTaster: pathogenic.

### Genome-wide detection of autozygosity regions and a novel *AP1S1* variant in patient 3

Based on the hypothesis of an autosomal-recessive secretory diarrhea due to a homozygous disease mutation inherited on a haplotype identical-by-descent, WES was performed and variant filtering prioritized within the 23 homozygous regions of > 4 Mb in size, identified in WES data from patient 3; and a homozygous *AP1S1* variant c.346G>A in a homozygous 37.0-Mb region on chromosome 7q was found, causing a glutamic acid to lysine change at codon 116 (g.100802394G>A; p.(Glu116Lys)). This variant was present in heterozygous state in her parents (Fig. 1d). The phenotype and variant information were submitted to the LOVD database (https://databases.lovd.nl/shared/individuals/). This variant was not listed in dbSNP, ESP, and EXAC databases, and was predicted to damage protein function [Polyphen2 score 1.00 (range 0–1), CADD score 34.0 (range 0–100)], PROVEAN score − 3.740 (deleterious), MutationTaster: pathogenic. Other rare or private variants with effects on protein function as determined by in-silico evaluation seen within coding regions or at splice sites are provided in Supplemental Table 1.

### Generation of an *AP1S1* knock-out enterocyte model

**Fig. 2:**
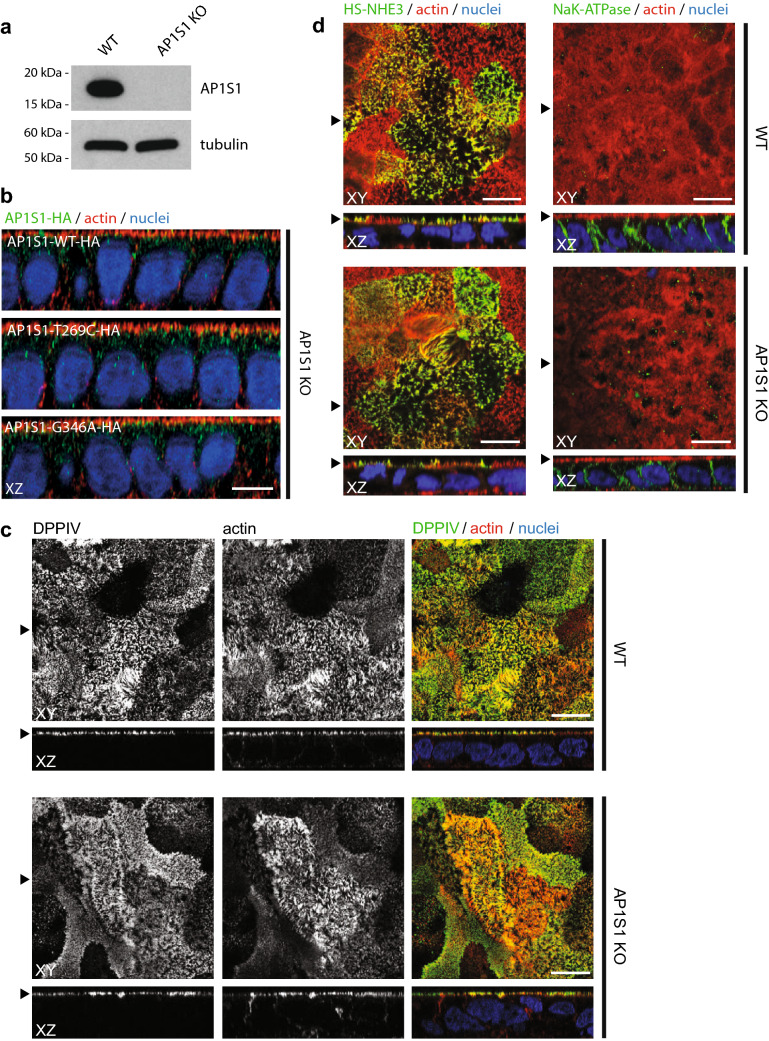
**a** Western blot detects AP1S1 in CaCo2 WT but not in CaCo2-AP1S1-KO cells. Tubulin is used as loading control. **b** Laser-scanning immunofluorescence micrographs (LSM) of CaCo2-AP1S1-KO cells expressing AP1S1-WT-HA, AP1S1T269C-HA or AP1S1-G346A-HA. Vesicular localization pattern of AP1S1. Scale bar 10 µm. **c** LSM of CaCo2 WT and AP1S1 KO cells. DPPIV staining, microvilli morphology (actin) and NHE3 apical localization (**d**) is unaffected by AP1S1 depletion. Basolateral localization of NaK-ATPase is not affected by loss of AP1S1. Scale bar 10 µm, arrowheads indicate respective plane in XY and XZ

To study an impact of the identified AP1S1 missense mutations on protein function, we generated an *AP1S1* knock-out (KO) enterocyte model cell line. CRISPR/Cas9 genome editing (Sanjana et al. 2014) was used to modify AP1S1 in the human CaCo2 enterocyte cell line to generate CaCo2-AP1S1-KO. Loss of AP1S1 was confirmed by Western blot analysis (Fig. 2a). A vector containing a guideRNA-resistant wildtype *AP1S1* cDNA tagged with *Influenza* hemagglutinin (AP1S1-WT-HA) was constructed. c.269T>C and c.346G>A point mutations were introduced into this construct with site-directed mutagenesis to generate AP1S1-T269C-HA and AP1S1-G346A-HA. All three constructs were re-expressed in CaCo2-AP1S1-KO cells by lentiviral transformation. Notably, the same vesicular subcellular localization pattern as for WT-AP1S1 was observed in CaCo2-AP1S1-KO cells expressing each AP1S1 mutant (Fig. 2b).

### Identified missense mutations do not rescue tight-junction and polarity abnormalities caused by loss of AP1S1

**Fig. 3:**
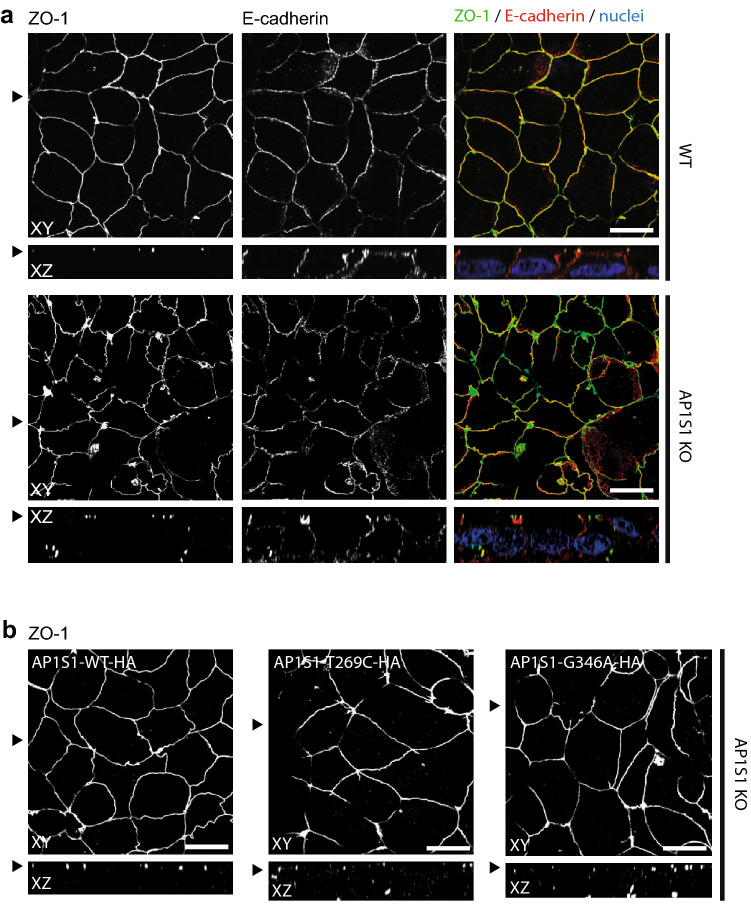
**a** Laser-scanning immunofluorescence micrographs of CaCo2 WT and AP1S1 KO cells. Tight-junction protein ZO-1 shows aberrant patchy localization upon AP1S1 deletion and in cells expressing patient mutations (AP1S1-T269C and AP1S1-G346A) but not AP1S1-WT (**b**). Basolateral E-cadherin staining is not affected by AP1S1. Scale bar 10 µm, arrowheads indicate respective plane in XY and XZ

**Fig. 4:**
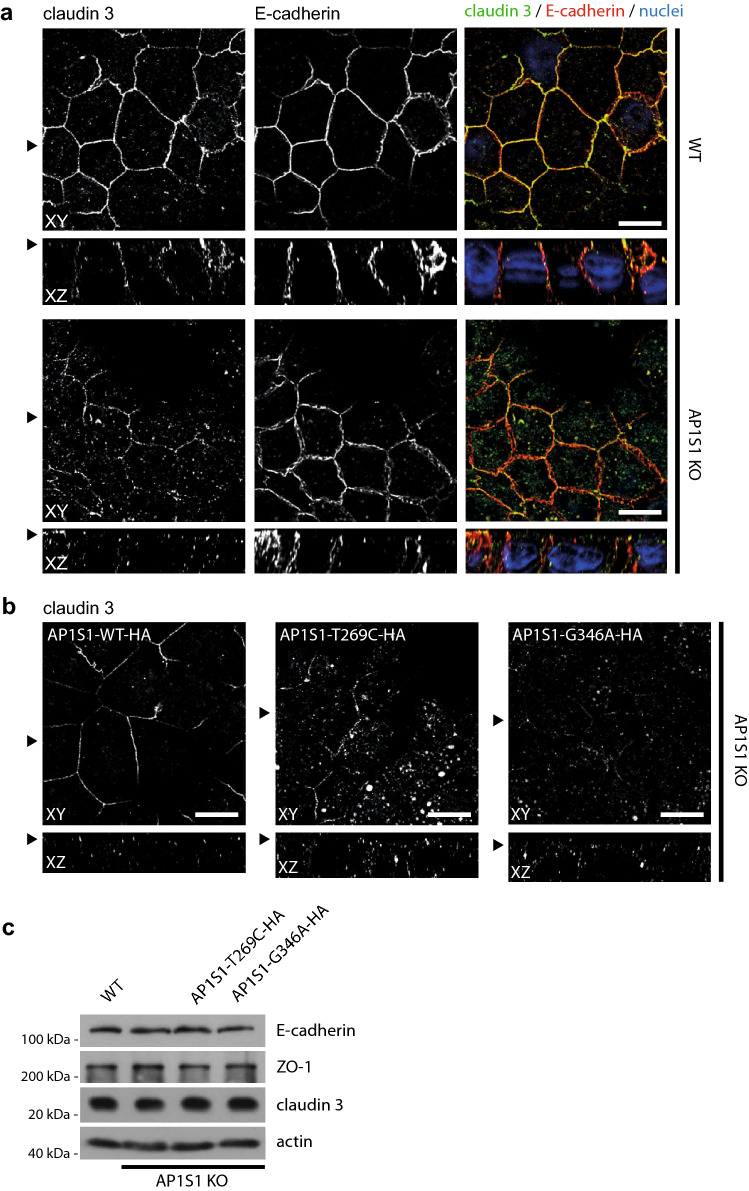
**a** Laser-scanning immunofluorescence micrographs of CaCo2 WT and AP1S1 KO cells. Tight-junction protein claudin 3 shows decreased signal at the apical circumference and increased intracellular localization upon AP1S1 depletion and in cells expressing patient mutations (AP1S1-T269C and AP1S1-G346A) but not AP1S1-WT (**b**). Basolateral E-cadherin staining is not affected by AP1S1 depletion. Scale bar 10 µm, arrowheads indicate respective plane in XY and XZ. **c** Western blot of E-cadherin, ZO-1 and claudin 3 in CaCo2 WT, AP1S1 KO and cells expressing patient mutations. Protein levels are not altered. Actin is used as loading control

**Fig. 5:**
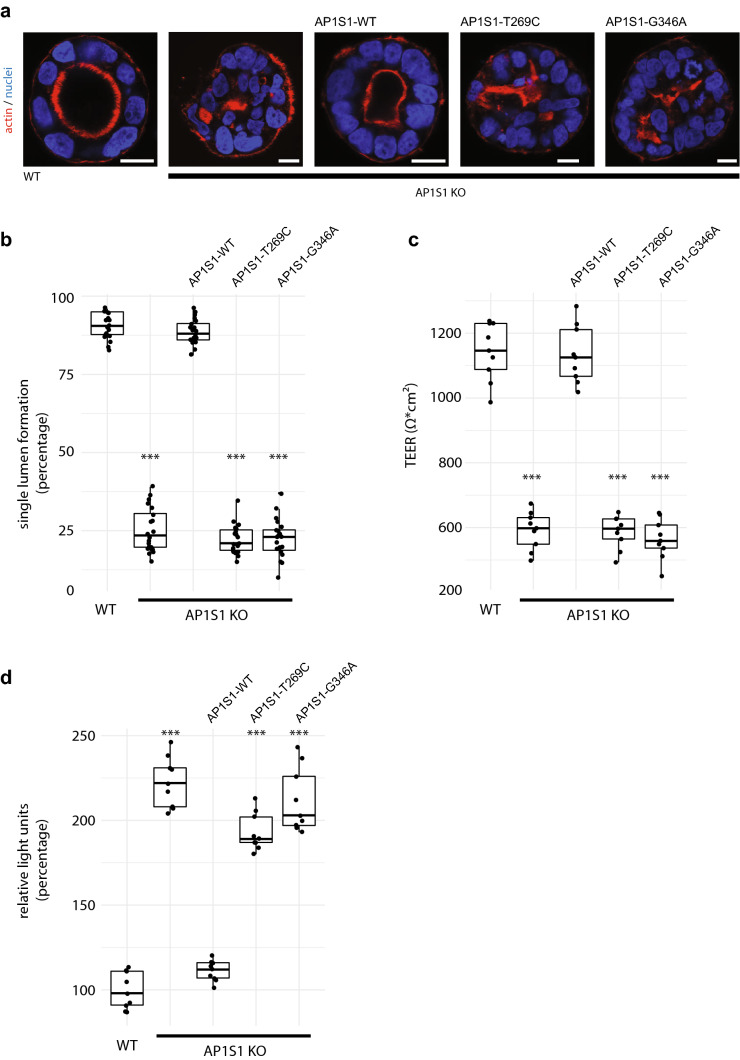
**a** Epithelial cysts formed by CaCo2 cells grown in Matrigel. Loss of AP1S1 or expression of mutated AP1S1 results in no or multiple lumina (actin staining) formation indicative of disturbed epithelial polarity. Scale bar 10 µm. **b** AP1S1 genotype-dependent central lumen formation (dot box plot, Mann–Whitney *U* test ***p < 0.001, three independent biological experiments per condition, a total of 150 cysts per condition were assessed). **c** Loss of AP1S1 without or with expression of patient mutations results in a significantly decreased transepithelial electrical resistance (TEER) in CaCo2 monolayers as compared with AP1S1 wild-type re-expression in CaCo2-AP1S1-KO cells (dot box plot, Mann–Whitney *U* test ***p < 0.001, three independent biological experiments per condition). **d** Epithelial barrier function for Dextran-TexasRed passage was tested by measuring fluorescence after ten hours in lower medium chamber. Increased fluorescence, e.g., increased monolayer permeability, was observed upon loss of AP1S1 or expression of patient mutations. Relative light units were normalized to WT measurements (dot box plot, Mann–Whitney *U* test ***p < 0.001, three independent biological experiments per condition)

The morphology of CaCo2-AP1S1-KO cells was assessed by laser scanning confocal immunofluorescence microscopy. 2D filter-grown CaCo2-AP1S1-KO monolayers displayed a properly formed actin filament cytoskeleton on the apical and basolateral plasma membrane including apical microvilli, comparable to the parental cell line (Fig. 2b). The localization of enzymes and transmembrane transporters on the apical plasma membrane, such as dipeptidyl peptidase IV (DPPIV) and sodium-hydrogen-antiporter 3 (NHE3), were unaffected by loss of AP1S1 (Fig. 2c, d). The basolateral sodium–potassium ATPase (NaK-ATPase) and the adherens junction protein E-cadherin were properly located (Figs. 2d, 3a). However, when compared with parental CaCo2 cells, the tight-junction protein zonula occludens 1 (ZO-1) showed rarefication at the apical circumference, marked accumulation at cellular contact sites and aberrant basal localization upon loss of AP1S1 in CaCo2 cells (Fig. 3a). This aberrant phenotype could be reverted by the stable reintroduction of AP1S1 wild-type protein (Fig. 3b). Expression of either AP1S1 missense mutation could not alleviate the observed phenotype and mislocalization of ZO-1 to basal plasma membrane remained (Fig. 3b). The findings obtained for ZO-1 were recapitulated with intestinal tight-junction marker claudin 3 that showed interruption of its normally marked circumferential localization (Barmeyer et al. 2015) (Fig. 4a, b). Yet, overall expression levels of ZO-1 and claudin 3 were unaltered (Fig. 4c). Although overall 2D monolayer polarity seemed unaffected, cyst formation and polarity were abnormal in 3D cultures of CaCo2 cells devoid of AP1S1 or expressing the AP1S1 missense mutants (Fig. 5a, b). Collectively, our data suggest that loss of AP1S1 causes aberrations in tight-junction protein localization and 3D epithelial polarity but does not affect apical plasma membrane composition; this phenotype cannot be rescued by AP1S1 missense mutants.

### Loss of AP1S1 disrupts epithelial barrier function, which cannot be rescued by the identified missense mutants

The observed mislocalization of tight-junction proteins ZO-1 and claudin 3 upon loss of AP1S1 prompted us to test barrier function in filter-grown CaCo2 monolayers. First, TEER was measured in CaCo2 monolayers. TEER was significantly reduced in CaCo2-AP1S1-KO monolayers compared with the parental cells, and could be reconstituted with reintroduction of WT, but not mutant AP1S1 (Fig. 5c). CaCo2 monolayer integrity was further investigated by determining dextran permeability. Low-molecular, fluorescently-labelled dextran was added to the apical compartment of the monolayer. The increases in fluorescence in the basal compartments of the culture dishes were measured after ten hours, and significant increases in permeability were observed for AP1S1 KO cells, and those expressing patient mutations (Fig. 5d). Both findings indicated that tight-junction formation was disrupted upon loss or mutations of AP1S1. Therefore, aberrant epithelial barrier function might underlie or contribute to congenital intestinal failure in patients with AP1S1 loss-of-function mutations.

## Discussion

Adaptor protein complexes orchestrate various intracellular endomembrane transport routes (Bonifacino 2014; Dell'Angelica and Bonifacino 2019; Park and Guo 2014). In polarized epithelial cells in particular, the adaptor complex (AP-1) controls basolateral transport, e.g., sorting of the transferrin receptor and the low-density lipoprotein receptor. These processes are carried out in dependence on certain cargo motifs and specific protein complex subunit compositions (Bonifacino 2014). AP-1 consists of 4 subunits: beta1, gamma, mu1 and sigma1 (Bonifacino 2014; Park and Guo 2014). Mutations in ubiquitously-expressed genes encoding subunits AP1B1 and AP1S1 cause MEDNIK syndrome (Alsaif et al. 2019; Montpetit et al. 2008). However, the role of AP1S1 in pathogenesis of MEDNIK-associated enteropathy is not yet completely understood.

Prior to our study, two disease-causing, homozygous *AP1S1* variants in seven patients were reported; these variants, c.364dupG and c.301-2A>G, caused premature stop codons and *AP1S1* mRNA decay (Martinelli et al. 2013; Montpetit et al. 2008), and were identified in each case with the full clinical pattern of MEDNIK syndrome, except for the variable presence of a peripheral neuropathy. Out of seven patients, three died at 2 and 18 months, and 27 years of age, and mental retardation (intellectual disability) was associated with cerebral atrophy.

Here, we extend the clinical and genetic spectrum of *AP1S1*-associated disease: we describe 2 novel *AP1S1* missense mutations in 3 patients, who died within the first 3 months of life due to an intractable diarrhea. Importantly, all other early symptoms of MEDNIK syndrome, i.e., deafness, ichthyosis or keratoderma, were not present.

To demonstrate that the identified missense mutations affect AP1S1 function, and to study AP1S1 loss-of-function in intestinal epithelium, we deleted AP1S1 from the intestinal CaCo2 cell line by CRISPR/Cas9 editing. As AP1S1 functions in polarized Golgi and post-Golgi trafficking (Bonifacino 2014), we hypothesized that trafficking of tight-junction and or tight-junction related proteins to the basolateral plasma membrane is disrupted upon loss of AP1S1. Indeed, we observed mislocalization of ZO-1, claudin 3 and disruption of lateral cell–cell junctions in our intestinal AP1S1-deficient cell line. We observed decreased TEER, increased epithelial dextran permeability and an abnormal cyst formation in 3D cultures of CaCo2 cells devoid of AP1S1. This indicated that tight junction abnormalities contributed to the observed disruption of epithelial barrier. While stable re-expression of wild-type AP1S1 in our model cell line completely reverted the observed phenotype in CaCo2-AP1S1-KO monolayers, whereas stable re-expression of either missense mutant did not, consistent with a loss of AP1S function caused by the missense mutations identified in this study.

Our study is the first to address the pathogenesis of the AP1S1-related enteropathy; rare cell biological studies on MEDNIK syndrome were based on the observation of elevated hepatic copper content in one such patient, and indicated that AP1S1 was essential for translocation of the copper transporter ATP7A to the plasma membrane (Martinelli et al. 2013), whereas copper transporter ATP7B localization was not affected (Overeem et al. 2019).

In transporting epithelia, tight junctions allow passive paracellular flux that contributes significantly to overall transepithelial absorption and secretion. Tight-junction integrity is essential for epithelial barrier function throughout the intestine. Decreased tight-junction protein expression as well as increased expression of leaky junction proteins, e.g., claudin 2, have been associated with epithelial leakiness and intestinal diseases (Barmeyer et al. 2015; Luettig et al. 2015). In particular, mice lacking claudins 2 and 15 die of malnutrition as a result of decreased paracellular cation flux and limited recycling of Na^+^ absorbed by transcellular pathways back to the lumen (Wada et al. 2013).

In AP1S1-related enteropathy, the disrupted intestinal epithelial barrier might result in increased intestinal ion and macromolecule loss thus explaining the observed secretory diarrhea. About 50 monogenic disorders that feature a severe congenital diarrhea have been described (Thiagarajah et al. 2018). The underlying pathophysiology varies from disrupted function or trafficking of apical ion transporters, e.g., NHE3 or CFTR (Barrett 2000; Janecke et al. 2015) to loss of enterocyte polarity by defects in cell adhesion proteins, e.g., EpCAM (Sivagnanam et al. 2008) or apical trafficking cascades, e.g., MYO5B, syntaxin 3 and syntaxin-binding protein 2 (Muller et al. 2008; Vogel et al. 2017; Wiegerinck et al. 2014). Of note, the apically residing antiporter NHE3 was not affected by the loss of AP1S1, and the formation of apical microvilli was normal. Unfortunately, a limitation of this study is the lack of intestinal biopsies from our patients and from MEDNIK patients to confirm tight-junction disruption as the direct cause of their enteropathy.

It is intriguing to speculate, that both missense mutations identified in this study, c.269T>C (p.Leu90Pro) and c.346G>A (p.Glu116Lys), disintegrate AP-1 complex stability. The same mechanism would apply to nonsense mutations in *AP1S1* and in *AP1B1*; however, the degree of spatio- or temporal disintegration of the complex caused by the identified missense and the nonsense mutations might be different; the missense mutations might represent hypomorphs that allow for residual complex function at least in the inner ear or skin. Such a distinction of potential hypomorphs and null mutations might have been missed with our cell biological work-up presented here. Whether mutations in further subunits of AP-1 will be identified as causing MEDNIK syndrome or non-syndromic enteropathy remains to be elucidated. Mutations in other adaptor protein complex subunits have been found in a number of rare neurologic phenotypes, suggesting that there is no redundancy of these proteins (Dell'Angelica and Bonifacino 2019). We add *AP1S1* to the list of congenital diarrhea genes, and show here that an epithelial barrier defect underlies the *AP1S1*-associated enteropathy.

## Electronic supplementary material

Below is the link to the electronic supplementary material.

Supplementary file1 (XLSX 39 kb)
